# Graph embedding on mass spectrometry- and sequencing-based biomedical data

**DOI:** 10.1186/s12859-023-05612-6

**Published:** 2024-01-02

**Authors:** Edwin Alvarez-Mamani, Reinhard Dechant, César A. Beltran-Castañón, Alfredo J. Ibáñez

**Affiliations:** 1 Engineering Department, Pontificia Universidad Católica del Perú, San Miguel, Lima, Peru; 2 Institute for Omics Sciences and Applied Biotechnology (ICOBA PUCP), Pontificia Universidad Católica del Perú, San Miguel, Lima, Peru; 3 Present Address: Calico Life Sciences, 1170 Veterans Blvd, San Francisco, CA 94080 USA; 4 Science Department, Pontificia Universidad Católica del Perú, San Miguel, Lima, Peru

**Keywords:** Graph embedding, Biomedical data, Biological network

## Abstract

Graph embedding techniques are using deep learning algorithms in data analysis to solve problems of such as node classification, link prediction, community detection, and visualization. Although typically used in the context of guessing friendships in social media, several applications for graph embedding techniques in biomedical data analysis have emerged. While these approaches remain computationally demanding, several developments over the last years facilitate their application to study biomedical data and thus may help advance biological discoveries. Therefore, in this review, we discuss the principles of graph embedding techniques and explore the usefulness for understanding biological network data derived from mass spectrometry and sequencing experiments, the current workhorses of systems biology studies. In particular, we focus on recent examples for characterizing protein–protein interaction networks and predicting novel drug functions.

## Introduction

In the literature, several reviews present graph embedding models used to solve multiple tasks such as pathogen-host protein interactions, predicting drug efficiency, linking a metabolite with a metabolic network, etc [1–3]. However, wide spread application of graph embedding techniques in the life-science community has been scarce, which may be in part because the complex mathematical framework underlying graph embedding requires considerable bioinformatical expertise. To make graph embedding known to a wider research community we have focused our review to be accessible for wet-lab biologists as well as bioinformaticians, mainly using more accessible wording for life scientists and focussing on potential future applications.

Biological data is usually presented as graphs; some of the most famous ones are represented in the book Cellular Biochemical Networks (Editor: Gerhard Michal), which describes the known metabolomic network of eukaryotic cells and comprises most of the cellular metabolites and their interactions (i.e., possible conversions and connections between metabolic pathways such as sugar and amino acid metabolism). Although traditional biology tools have been extremely successful in identifying most components and some of the major linear interactions contained in the Cellular Biochemical Networks graphs, one of the significant challenges in biology is comprehending the nonlinear or dynamic interactions among the cellular constituents to unravel the organization and interactions within cellular networks. For example, understanding which metabolic subnetworks are active in a particular cell type under specific conditions is critical to decipher the influence of the metabolic network on cellular function.

Mass spectrometry (MS) is an excellent example of a tool for understanding the underlying interactions among large numbers of cellular constituents. MS-based metabolomic and proteomics studies can follow various linear and nonlinear interactions (based on signal abundances) and dynamic interactions from time series measurements. The interactions are visualized via correlation plots of the MS signals [4, 5]. In a correlation plot, metabolites, proteins, etc., are represented as dots (or nodes), and a line illustrates their correlations with other network elements. Using carefully designed experiments and bioinformatic tools makes it possible to model and quantify the different types of interactions between the nodes. Hence, a traditional approach in molecular biology is to compare two or more graphs to identify which metabolites or proteins in the biological network are associated with a particular physiology (i.e., disease) or phenotype of interest [4, 5].

Unfortunately, clear insight into biological information via visual inspection of the correlation plots is challenging due to the large number of biological species present in cells that MS can detect. Furthermore, artifacts such as the presence of ghost peaks or batch effects can futher obscure the information within these graphs [4, 5]. Graph embedding techniques have been developed to analyze complex graphs of diverse origins. A graph embedding technique takes graphs as input and converts the graphs into a matrix of vectors (i.e., a lower-dimensional latent space), thus allowing researchers to better identify the interactions between their different elements. Although graph embedding techniques have been applied to various fields of study, e.g., to analyze relationships between client and providers in financial transactions [6], to recommend locations using recommender systems [7], or to detect malware [8]; they have not been routinely applied to biological systems and are not well-known to life-scientists.

This review discusses the suitability of graph embedding techniques for analyzing masss spectrometry- and sequencing-based biomedical data and explains the theoretical background to understand graph embedding. We classify graph embedding techniques from the perspective of biomedical data, considering the canonical classification, thereby subdividing graph embedding techniques into random walk-based, matrix factorization-based, and deep learning-based algorithms. Additionally, we review articles that applied graph embedding for link prediction, node classification, and node clustering tasks on biomedical data and highlight novel biological insights obtained by graph embedding. In particular, we will focus on protein–protein and drug–protein interactions. Our review will help future readers to identify, which graph embedding models can be applied to solve a given task on biomedical datasets, which datasets can be used, and which metrics are available to evaluate the results.

The paper is structured as follows: section “Theory of graphs embeddings” contains the necessary definitions and summarizes the theoretical background of graph embedding. Then, section “Applications of graph embeddings in mass spectrometry- and sequencing-based biomedical data” describes the existing applications of graph embedding techniques on biomedical data. Finally, section “Conclusion” discusses conclusions and future applications.

## Theory of graphs embeddings

### Background techniques

To be able to understand graph embedding, we first must introduce the term word embedding, which transforms a group of words (i.e., text) into a matrix of vectors and is frequently used in natural language processing (NLP) [9]. In more detail, word embedding technique results in the (n-dimensional) vector representation of a word (token) within a text [10]. Since words often occur in the same semantic or syntactic context, a cosine similarity measure among the vectors in the matrix can be used to identify the relationship between words. Hence, the semantic and syntactic similarity between words can be mathematically identified [11, 12]. For example, word embedding is used when a word processing program suggests a phrase after the computer user types just a few words. Two different strategies were proposed for word embedding (i.e., architectures): Continuous bag-of-words (CBOW) [13] predicts a word $w_i$ in one particular position in the sentence based on the context of words surrounding that position $w_{i-2}, w_{i-1}, w_{i+1}, w_{i+2}$, while continuous skip-gram model [13] predicts the context (surrounding words) with respect to a particular word in the sentence. The first formulation of skip-gram model defines the conditional likelihood $P(w_{context} \mid w_{center}) \approx P(w_{o} \mid w_{c})$ utilizing the function softmax [13, 14],

1 $$\begin{aligned} P(w_{o} \mid w_{c}) = \dfrac{exp(u_o ^\top v_c)}{\sum _{i=1}^{\mid W \mid } exp(u_i ^\top v_c)} \end{aligned}$$

where *o* is the index of the context word (output) in the dictionary, *c* is the index of the central word (input) in the dictionary, and *W* is the vocabulary.

Similarly, *continuous bag-of-words* defines conditional likelihood $P(w_{c} \mid w_{o_{1}},\ldots , w_{o_{2m}})$ [13, 14], where $o_{1},\ldots , o_{2m}$ are the indexes of the context words in the dictionary.

2 $$\begin{aligned} P(w_{c} \mid w_{o_{1}},\ldots , w_{o_{2m}}) = \dfrac{exp \big (\dfrac{1}{2m} u_o ^\top (v_{o_{1}} +\cdots + v_{o_{2m}})\big )}{\sum _{i=1}^{\mid W \mid } exp \big (\dfrac{1}{2m} u_i ^\top (v_{o_{1}} +\cdots + v_{o_{2m}})\big )} \end{aligned}$$

Although the skip-gram architecture performs slightly worse on syntactic tasks than the CBOW model, it does much better on semantic tasks [13]. Executing the definition (Equ. 1) has a very high computational cost [13, 14]. Therefore, [15] optimized the training process of the skip-gram model by adding the hierarchical softmax and negative sampling techniques.

Graph embedding is applied to dot (scatter) graphs. In analogy to *word embedding*, in *graph embedding* a point (i.e., node) in a graph is considered as a word, which is surrounded by other points (i.e., words). Furthermore, the graph contains information about the relationship between any two given points (words); this relationship is defined as an edge between two nodes. Hence, graph embedding can be used to create a matrix of vectors for all the nodes in a graph based on their edges by using the following analogy [16–19]: given a sequence of words, $S_1^n = (w_1, w_2,\ldots , w_n)$ where $w_i \in W$, it can be inferred $P(w_n \mid w_1, w_2,\ldots , w_{n-1}) \approx P(v_i \mid v_1, v_2,\ldots , v_{i-1})$ and $v_i$ represents a node in a graph *G*.

### Graph embedding

The following definitions are useful to better understand and develop graph embedding and its applications.

#### Definition 1

**Fig. 1:**
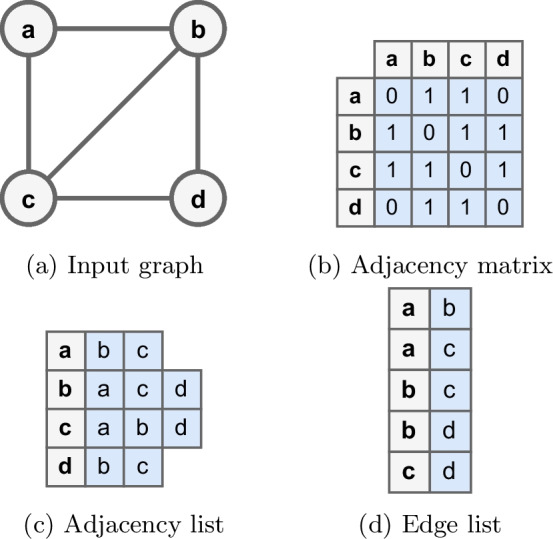
Graph representation

(Graph) In mathematics and computer science, a graph is a scatter plot with a defined data structure. Let *G* be a graph, defined as $G = (V, E)$ where $V = \{v_1, v_2,\ldots , v_n\}$ is a set of nodes (vertices), and *E* represents the connection (edge) between 2 nodes $(v_i, v_j) \in V$ [1, 20–23]. Given a graph (Fig. 1a), this graph can be represented by an adjacency matrix: is 1 when there is an edge from node $v_i$ to node $v_j$, and is 0 when there is no edge (Fig. 1b). The adjacency list groups the neighboring nodes of each node $v_i$ (Fig. 1c), while the edge list consists of ordered pairs $(v_i, v_j)$ when there is an edge from node $v_i$ to node $v_j$ (Fig. 1d) [20, 21, 24, 25].

#### Definition 2

(Homogeneous and heterogeneous graphs) In a homogeneous graph, all nodes and/or edges are of the same type. For example, in the friends’ network, each node represents a person, and an edge represents friendship between two people. In contrast, in heterogeneous graphs, nodes and edges can be of different types. Heterogeneous graphs are exemplified by an education network, in which there may be nodes representing teachers and students, and it is possible to have the relationships (edges) between teachers (colleagues), between teachers and students, and between students (classmates) [1, 20, 21, 24]. By their nature, biochemical networks can be defined as homogeneous or heterogeneous graphs. For example, protein–protein interaction studies are represented in homogeneous graphs [26–29], while miRNA-disease/gene interaction studies are represented by heterogeneous graphs [30–32].

#### Definition 3

(Directed and undirected graphs) In directed graphs (digraph), the list of nodes (i.e., vertices) that generates the graph is ordered, and each interaction (i.e., edge) has a direction. Traversal in this type of graph is done according to the direction of the interactions among nodes, while in undirected graphs traversal can be done in both directions [1, 20, 21, 24]. In metabolic pathways, both types of graphs are present. Metabolic pathways, in which each product (i.e., node) is solely dependent on its precursor (i.e., a previous node in the pathway), can be defined as directed. However, most metabolic pathways are represented as undirected graphs, since their chemical reactions are reversible and regulated by feedback loops, where downstream products influence the formation of their upstream precursors (e.g., in glycolysis) [33, 34].

#### Definition 4

(First-order and second-order proximity) The first-order proximity measures the proximity between a pair of nodes $v_i$, $v_j$, and represents the weight *w* of the edge $e_{ij}$ ($w \ge 0$). If the edge does not have a weight, then the default value is 0. Then, first-order proximity is defined as the neighborhood of the node $v_i$ containing a set of adjacent nodes $N_{v_i} = \{v_k \mid e_{ik} > 0, k \ne i\}$. The second-order proximity measures the number of 2-hop paths between a pair of nodes $v_i, v_j$ [2, 24].

#### Definition 5

**Fig. 2:**
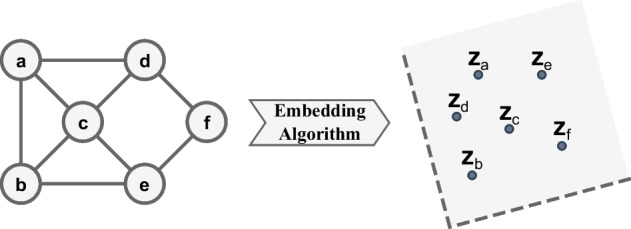
Graph embedding scheme

(Graph embedding) Given a graph as input $G = (V, E)$, graph embedding (see Fig. 2) is defined as a mapping function $f: v_i \rightarrow Z_i \in {\mathbb {R}}^{d}$ (latent space) with $i \in \{1, 2,\ldots , n\}$ where $d \ll$ |*V*| and $Z_i$ is a vector of dimension *d* known as an *embedding* [2, 22, 24].

### Classification of graph embedding techniques

Most commonly, graph embedding techniques are classified as either matrix factorization-based, random walk based, or deep learning-based [1, 2, 22–24, 35].

However, in the literature, an alternative classification has been introduced based on the point of view of the mathematical problems, which can be *vector point-based*, *gaussian distribution-based*, or based on *dynamic graph embedding* [1]. Vector point-based approaches aim to project the nodes of a high-dimensional graph onto low-dimensional vectors within a vector space [1]. Gaussian distribution-based methods allow the vector representation (embedding) of a node as *potential functions* of *continuous densities* in a vector space. [1]. Dynamic graph embedding is often the method of choice for practical applications, as many networks are dynamic and evolve, leading to the addition of removal of nodes or edges [1].

Alternatively, it was proposed that embedding techniques can be grouped from the perspective of biomedical networks, including biomedical relation data, biomedical knowledge graphs, biomedical ontology, or clinical data, in *non-attributed network embedding* and *attributed network embedding* [36]. Below is the classification of non-attributed network embedding [1, 2, 22, 24, 35, 36] and attributed network embedding [2, 36]. Table 1 shows the graph embedding models published by category.

**Table 1:** Network embedding models

Category	Publications
*Non-attributed network*	
Shallow embeddings	[16, 17, 19, 37–53]
Graph neural networks	[54–62]
*Attribute network*	
Semantic matching models	[63–78]
Translational distance models	[79–85]
Meta-path-based methods	[18, 86–90]

### Mathematical concepts behind graph embeddings

Shallow embeddings are the earliest graph embedding technique applied to life-science data based on homogenous networks (i.e., networks based on only one biological entity, such as proteins). Shallow graph embeddings are subdivided into random-walk and matrix-factorization algorithms. Examples of random-walk algorithms are (DeepWalk [16] and Node2vec [17]; while matrix-factorization examples are graph factorization [43] and GraphRep [44].

**DeepWalk** [16] was the first graph embedding technique used to represent the vertices (nodes) of a homogeneous graph in vectors [91]. The process begins when the random walk algorithm generates a sequence of vertices. The model is then trained using the skip-graph algorithm [13]. Finally, the result is the vector representation for each vertex, also called embedding.

**Fig. 3:**
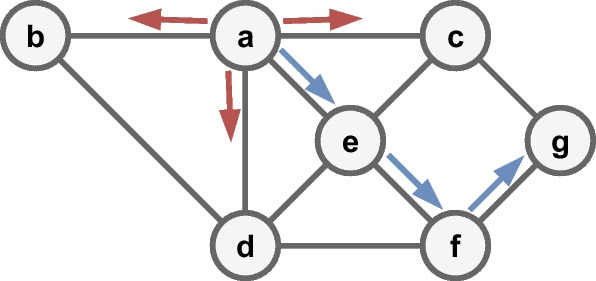
BFS (red arrows) and DFS (blue arrows) traversals, from node *A* with a path length of 3

**Node2vec** [17] is a generalization of DeepWalk [16]. The authors added two parameters, *p*, and *q*, which drive the generation of paths (see Fig. 3) by using the idea of breadth-first traversal (BFS) and depth-first traversal (DFS). When $q > 1$, the traversal approaches BFS, and the random walks lead to a *micro-view* of node neighborhoods. In contrast, $q < 1$ is an exploration *macro-view* that approximates a DFS traversal for node neighborhoods [1]. The authors of the base article used the values of $p=1$, $q=2$ for a micro-view and the values of $p=1$, $q=0.5$ for a macro-view. The parameters *p* and *q* also control how fast a path is explored, and the neighborhood of an initial node $v_i$ is left. The authors performed multi-label classification and link prediction experiments to verify their proposal. Results were evaluated using the F1-score metric.

While Deepwalk and Node2vec provided a solution to tasks such as link prediction, node classification, node clustering (community detection), and visualization, two random-walk algorithms, Netpro2vec [92] and Pathway2vec [33], were proposed to better analyze biomedical datasets.

**Netpro2vec** [92]: In the techniques described above, nodes of a network were transformed into tokens. Instead, the main concept of Netpro2vec is to transform networks into documents. The process is carried out in 3 steps: 1) building the probability distributions representing each graph, 2) extracting tokens from probability distributions, and 3) building the graph embedding using token extraction. The graph is then represented as a word document (a set tokens), and the Doc2vec (document embedding) technique is applied to obtain the graph embedding [93]. The proposal was compared with other techniques of whole-graph embeddings to solve classification tasks in gene networks. The results were evaluated based on accuracy, precision, recall, F-measure, and Matthews correlation coefficient (MCC) metrics.

**Pathway2vec** [33] incorporates multiple random walk-based techniques, Node2vec [17], Metapath2vec, Metapath2vec++ [18], JUST [94], and RUST [33], to represent learning by automatically generating features of metabolic pathways. It consists of three layers that interact: compounds, enzymes, and pathways. This interaction between layers results in a heterogeneous network of multi-layer information, and each layer has associated nodes. The layered architecture captures meaningful relationships to learn a low-dimensional space based on neural embeddings of metabolic features. Finally, applying the skip-gram [13] model, the embeddings for each node are extracted. Pathway2vec was applied for node clustering, embedding visualization, and pathway prediction tasks. Evaluation of the results was performed using MetaCyc software and F1-micro metric.

**Graph Factorization (GF)** [43]: GF is a factorization technique based on partitioning a graph to minimize the number of neighboring vertices instead of edges between partitions. GF begins from the assumption that the information regarding the presence of an edge (*i*, *j*) with a weight $Y_{ij}$ can be captured by the inner product between vertices with attributes $\langle Z_i, Z_j \rangle$. Finally, the value of the vector Z is determined by the following objective function:

3 $$\begin{aligned} f(Y, Z, \lambda ) = \dfrac{1}{2} \sum _{(i, j) \in E} (Y_{ij} - \langle Z_i, Z_j \rangle )^2 + \dfrac{\lambda }{2} \sum _{i} \Vert Z_i \Vert ^2 \end{aligned}$$

where $\lambda$ is the regularization parameters, *E* is the list of edges. To validate their proposal, the authors applied GF on a graph of 200 million vertices and 10 billion edges. In order to evaluate convergence and execution time, they used 3 architectures: a single machine, a synchronous parallel implementation and an asynchronous parallel implementation. The results showed that asynchronous parallel implementation is very beneficial for scalability.

**GraRep** [44]: GrapRep is a model for learning node representation. This model captures the relational information of different k-steps with different values of k between vertices of the graph, directly manipulating different global transition matrices defined on the graph without slow and complex sampling processes. GraRep defines different loss functions and optimizes each model with matrix factorization techniques, constructing global representations of each node by combining the different model representations. Experiments were run to solve the node clustering and node classification tasks on linguistic networks and social networks, respectively. In both tasks, GraRep showed an empirical efficiency of the learned representations compared to the LINE and DeepWalk models.

While shallow-embedding algorithm applications focus on solving link prediction, node classification, and community detection tasks, more complex problems such as graph matching, subgraph matching, and calculating the maximum common subgraphs require more complex models. Graph-neural network (GNN) algorithms can address these problems by combinatorial optimization using graph theory. Furthermore, these problems are solved through representation learning (deep learning); for example, in [95] a GNN model is proposed that addresses the subgraph matching problem for molecular fingerprint detection.

**Graph Convolutional Network (GCN)**: Kipf et al. [56] present GCN for semi-supervised learning that works directly on graphs. GCN is a variation of convolutional neural networks. It scales linearly with the number of edges and encodes the local structure of the graph and features of nodes. The task of node classification is approached on a graph with partially labeled nodes, using a neural network *f*(*X*, *A*) trained in a supervised environment with node feature matrix *X* and adjacency matrix $A \in {\mathbb {R}}^{N \times N}$. For this purpose, a multilayer GCN is considered with the following layer-wise propagation rule.

4 $$\begin{aligned} H^{(l + 1)} = \sigma (\tilde{D}^{-\frac{1}{2}} \tilde{A} \tilde{D}^{-\frac{1}{2}} H^{(l)} W^{(l)}) \end{aligned}$$

where $\tilde{A} = A + I_N$ is the adjacency matrix of undirected graph with added loops, $I_N$ is the identity matrix, $\tilde{D}_{ii} = \sum _{j} \tilde{A}_{ij}$ and $W^{(l)}$ is a layer-specific trainable weight matrix, $\sigma (.)$ is an activation function, $H^{(l)} \in {\mathbb {R}}^{N \times D}$ is the matrix of activations in the $l^{th}$ layer $H^{(0)} = X$. The experiments were run on 4 datasets (Citeseer, Cora, Pubmed, and NELL), and the results showed that CGN significantly outperforms DeepWalk.

In the case of attribute data—biomedical data based on heterogeneous networks (i.e., networks based on more than one biological entity, such as drug–protein target interactions)—the graph embedding algorithms must consider both the node distribution and the edge information of the graph. Embeddings are generated that encode the proximity between nodes based on their attributes and connectivity patterns. Graph embeddings algorithms for attribute data can be divided into semantic matching models (e.g., DDKG, DistMult, etc.), translational distance models (e.g., TransE, TransR), and meta-path-based methods (e.g., Metapath2vec).

**DDKG**: Xiaori et al. [96] used an approach denominated “attention-based knowledge graph representation learning framework” or DDKG to simultaneously consider drug attributes and triple facts in knowledge graphs (KG). A triple fact is the link between one entity (e.g., metabolite, protein, etc.), usually referred as subject or head, and another entity referred as object or tail. The relationship between this two entities is referred as relationship or label. Xiaori et al.’s work aimed to use all the information available in biomedical KGs and improve the results in the link prediction task in drug–drug interaction (DDI) networks. The proposal was developed in 4 steps: 1) Building the KG, 2) Generating the initial embeddings for each drug according to its KG, 3) Generating the global embeddings of the drugs considering the node-embeddings of their neighbors, 4) finally, DDKG determines the probability of interaction of drugs in pairs with their respective embeddings through a binary classification. The experiments were conducted on two biomedical KGs and compared with ten state-of-the-art models, including LINE and SDNE. Results obtained from DDKGs were evaluated by metrics of accuracy, sensitivity, specificity, AUC, and AUPR, demonstrating that DDKGs outperformed the state-of-the-art models.

**DistMult** [67] considered learning entity and relationship representations in knowledge bases (KBs) using the neural-embedding approach. The learning process seeks to learn entity and relationship representations such that valid triple facts (i.e., known facts) receive high scores. The triple facts are denoted by ($e_1$, *r*, $e_2$), where $e_1$ is the subject, $e_2$ is the object, and *r* is the relationship between the two. The first layer of the model projects a pair of entities from the input into low-dimensional vectors, and the second layer combines these two vectors into a scalar to be compared by a scoring function. Entity representation learning can be defined as:

5 $$\begin{aligned} y_{e_1} = f(W X_{e_1}), \quad y_{e_2} = f(W X_{e_2}) \end{aligned}$$

where *f* can be a linear/nonlinear function, *W* is a parameter matrix, *W* can be initialized randomly/pre-trained, and *X* is a one-hot/n-hot vector representing the input entities $e_1$ and $e_2$. DistMult was empirically evaluated for link prediction tasks on the Freebase dataset. The results showed that a bilinear model successfully captures the compositional semantics of the relationships. It is also reported that DistMult outperforms TransE with a top-10 accuracy of 73.2% versus 54.7%.

**TransE**: Antoine et al. [80] addressed the problem of embedding different class entities (e.g., metabolites, proteins, etc.) and relationships of multi-relational data in low-dimensional latent spaces. The primary condition is that all the different entities (e.g., protein, metabolite, gene, etc.) must be present in a directed graph. In this directed graph, a triple fact consists of one entity (designated head), which is related to another entity (designated tail) by an edge (designated label). TransE is an energy-based model that learns embeddings of low-dimensional entities. For TransE the relationships are represented as translations in latent space; if a strong relation (edge) exists among two nodes (i.e., head and tail), then the embedding of the tail entity must be similar to the embedding of the head entity plus some vector that satisfies the relationship. For its simplicity, TransE has a small number of parameters and is scalable. Experiments showed that TransE performs well and significantly outperforms the RESCAL method in the link prediction task on two large knowledge base, Firebase and Wordnet.

**TransR** [82]: In contrast to the TransE model, where entities and relations (edges) are embedded in the same latent space, in TransR it was proposed to build the embeddings of the entities and the edges in separate latent spaces linked by specific relation matrices, yielding one entity space and multiple relation spaces. TransR was based on the idea that entities that have a relationship of the form (head, label, tail) are first projected from the entity space into the r-relation space as $h_r$ and $t_r$ with $M_r$ operation, and then $h_r + r \approx t_r$. The relation-specific projection can make the head/tail entities that actually hold a strong relation (edge) close to each other and also move away those that do not. In the experiments, Lin et al. [82] evaluated the model with three tasks: link prediction, triple classification, and relational fact extraction using the WordNet and Freebase datasets. The results showed that TransR obtains significant improvements compared to TransE. Additionally, they proposed CTransR, a combination of TransR and Clustering.

**Metapath2vec** [18]: Unlike DeepWalk [16] and Node2vec [17], Metapath2vec, guides and generates paths using random walks through meta-path schemes. It captures the structural and semantic relationships between different types of nodes in heterogeneous networks. Formally, a meta-path is defined as a path ${\mathcal {P}}$ represented by, ${\mathcal {P}}: V_1 \xrightarrow {R_1} V_2 \xrightarrow {R_2}\ldots V_t \xrightarrow {R_t} V_{t+1}\ldots \xrightarrow {R_{l-1}} V_l$, where $R = R_1 \circ R_2 \circ \cdots \circ R_{l-1}$ defines complex relationships between node types $V_1$ and $V_l$. The skip-gram architecture is also used by Metapath2vec to determine embeddings. Dong et, al. [18] evaluated their proposal on heterogeneous graphs for solving multi-classification nodes, node clustering, and similarity search problems. The results were evaluated using the F1-score metric.

## Applications of graph embeddings in mass spectrometry- and sequencing-based biomedical data

Applications of graph embedding techniques for mass spectrometry- and sequencing-based data covered in this review are summarized in Table 2 [26, 31, 33, 92, 97, 98]. By their nature, certain—OMICs data can be stored in a graph data structure. For example, gene–gene, protein–protein, and metabolite-metabolite interactions can be stored in homogeneous graphs. In contrast, heterogeneous graphs can contain multiple species, e.g., drug–protein, gene–protein interactions, etc. and analyzing these graphs can contribute to biological knowledge. However, computational tools to study graph data structures in biological graphs can suffer from high computational and space costs, especially in large-scale information containing graphs [28]. Graph embedding algorithms can then be used to identify interactions between heterogeneous nodes such as: drug–target [26, 99–101], miRNA-disease [30, 31], miRNA-target [32], miRNA-gene [32], microbe-drug [102], gene–disease [31, 103], gene–pathway [31], cell–gene [104], chemical–disease [31]. On the other hand, the interaction between homogeneous nodes may be protein–protein [26–29], drug–drug [34, 100, 102], microbe-microbe [102], gene–gene [104].

As an example, Su et al. [28] applied graph embedding to improve the identification of protein–protein interactions. To avoid the high computational cost of identifying the possible protein–protein interactions based on previous graph embedding techniques, the authors studied different approaches (algorithms) to accelerate graph embedding and improve its accuracy. The authors’ contribution was 2-fold. Firstly, their approach denominated LPPI integrated protein attributes into the graph embedding task. This way, multi-view information was used, improving the accuracy of the graph embedding process. Secondly, the graph was reconstructed using the GraphZoom algorithm to reduce the graph’s size. Therefore, the authors could accelerate the efficiency of the embedding algorithms. Combining the above two aspects, the authors’ algorithm, LPPI, saves execution time without losing accuracy (AUC 0.99996) in identifying protein–protein interactions in a large dataset.

Despite representing a major advance in the use of graph embedding, Su et al. [28] only used a homogeneous dataset from protein data. However, biological information from systems biology studies is typically derived from multi-omics datasets and contain heterogeneous information (DNA, RNA, protein, and metabolite information). Furthermore, as the network of interactions is time or condition-sensitive, multilayer networks must be considered [4].

Gong et al. [105] proposed the use of a multilayer network embedding to handle data sets with multiple types of nodes and edges found in heterogeneous graphs. This approach becomes extremely useful for evaluating the performance of node embedding in link prediction, which tries to predict edges that most likely will appear in theoretical networks (not experimentally measured data); this is similar to the approach performed by bioinformatics in in-silico studies. As some tested datasets are very large and complex, it is hard to predict links on the whole node sets. Hence, Gong et al. [105] suggested first extracting a core set of nodes of each dataset and conducting link prediction in these core sets. Hence, many authors similar to Gong et al. are encouraging the use of more complex graph-embedding algorithms that are based on combinations of the above-mentioned ones. These combinations of graph-embedding algorithms are known as encores or graph neural networks.

For example, Ray et al. [106] used a combination of graph-embedding algorithms as proposed by Gong et al. to generate a graph embedding encore algorithm approach to identify potential drugs that could affect the protein–protein interaction (PPI) between the SARS-CoV-2 virus and its human target proteins. The SARS-CoV-2 viral protein and human interaction datasets (i.e., protein interaction graph) were based on the experimental data obtained by Gordon et al. [107] by means of affinity-purification mass spectrometry (AP-MS) screening and on the theoretical data by Dick et al. [108].

The graph embedding-based algorithm proposed by Ray et al. [106] to repurpose drugs against COVID-19 considered that the available data was heterogeneous. They suggested to combine three different data sets: (i) SARS-CoV-2—host protein interactions, (ii) human protein–protein interactions, and (iii) drug–human protein interactions to predict possible novel treatments to interfere with infection. As described by Gong et al. [105], these three datasets were very large and complex; hence, Ray et al. [106] had to reduce the dataset complexity by performing the data reduction step, i.e., a first graph embedding based on the Nod2vec algorithm to obtain the feature matrix ($\texttt{X}$). In the second step, the novel graph embedding algorithm denominated variational graph autoencoder (VGAE) was used for link prediction tasks. As input, VGAE receives the adjacency matrix ($\texttt{A}$) and the feature matrix ($\texttt{X}$) from the original graph ($\texttt{X}$ replaces the one-hot matrix that the VGAE model uses by default and also helps improve prediction precision). The encoder of VGAE converts the input data to lower-dimensional representation ($\texttt{Z}$) and the decoder takes $\texttt{Z}$ to reconstruct the original input in ($\hat{\texttt{A}}$), where $\hat{\texttt{A}}$ is similar to $\texttt{A}$, and in $\hat{\texttt{A}}$ new connections between the different types of nodes can be discovered.

The results of Ray et al. [106] were compatible with those observed by other authors. For example, Ray et al. [106] identified the angiotensin-converting enzyme-2 (ACE-2) as a potential drug target against SARS-CoV-2 [109, 110]. Interestingly, the authors also found that drugs used to prevent Malaria and pneumocystis pneumonia (PCP) relapses, such as Primaquine, have therapeutic potential against SARS-CoV-2 based on the interaction of Primaquine with the TIM complex, consisting of TIMM29 and ALG11.

Similarly, Zitnik et al. [111] used a graph convolutional network, a combination of graph-embedding algorithms with a convolutional neural network that can work directly on graphs, to predict clinical side effects in patients taking multiple drugs simultaneously.

As in the case of Ray et al. [106], Zitnik et al. [111] combined multimodal graphs of protein–protein interactions, drug–protein target interactions, and known clinical drug side effects. Their new graph embedding algorithm, named Decagon, could accurately predict drug side effects in patients with complex diseases or co-existing conditions necessitating simultaneous medication for their treatment.

The use of shallow embeddings, such as (Nod2vec) is limited as shallow embeddings do not share information between the nodes and do not take advantage of the characteristics of the nodes in the coding process. To mitigate these limitations, graph neural networks (GNN) have more sophisticated encoders that take advantage of the structure, features, and attributes of graphs [112].

Su et al. [113] proposed constrained multi-view nonnegative matrix factorization (CMNMF), a model based on GNN, to determine the similarity between drugs and viruses within their space of characteristics (latent space). Therefore, CMNMF is oriented towards preserving drug and virus similarity information as much as possible. Then, they apply a graph convolutional network (GCN) with attention-based neighbor sampling to optimize the vectorial representation of drugs and viruses in virus-drug associations (VDA) networks, whereas VDA networks are considered heterogeneous graphs. The experiments were executed on three VDA datasets to identify possible drugs against SARS-CoV-2. The embedding algorithm from Su et al. outperformed other models and was evaluated with the accuracy, F1, AUC, and AUPR metrics.

Decagon, a DeepWalk neural graph embedding, outperformed baseline algorithms by up to 69% (accuracy). Specifically, Decagon could automatically predict side effects with a known strong molecular basis with high precision, but still performed well on predicting side effects with a non-molecular basis due to its effective sharing of model parameters across edge types.

Finally, Nelson et al. [114] mentioned the advantages of graph embedding techniques compared to other techniques that operate directly on biological/biomedical networks. One advantage is a more rapid analysis of the learnt embedding. Unlike the tasks mentioned in the other works (link prediction, node classification, and node clustering), Nelson et al. [114] demonstrated the usefulness of graph embeddings for more specific tasks in biology, such as protein network alignment, protein module detection, and protein function prediction. Taken together, these examples establish the high value of graph embedding techniques for the analysis of mass spectrometry—and sequencing-based—OMICs datasets. Several other applications have been published, which could not be discussed in greater detail, but have been showcased in Table 2 and classified for the use for (i) link prediction, (ii) node classification, and (iii) node clustering tasks.

**Table 2:** Summary of graph embedding on biomedical data

**Techniques**	**Dataset**	**Applications**	**Evaluation Metrics**
Combined DeepWalk, LINE, Node2vec, and SDNE [26]	MATADOR, PubTator, and BioGRID	Link prediction	AUC, AUPR, MAP, Avg. R-precision, and Precision@k
HeteWalk [115]	HPRD, MISIM, MimMiner, DisGeNET, and miRTarBase	Link prediction	AUC
Cascade model [97]	BioChem, Drug Bank, and PubChem	Link prediction	Accuracy, hits@10, and AUC
[29]	Krogan, Dip, and BioGRID	Node clustering	Precision, recall, F-score, fraction, geometry accuracy, and MMR
HNERMDA [102]	MDAD and aBiofilm	Link prediction	Accuracy, AUC, and AUPR
PmDNE [30]	HMDD3.0	Link prediction	AUC, AUPR, precision, accuracy, recall, and F1-score
HO-VGAE [27]	HI-II-14, HI-III, Lit-BM-13, BioGRID, and Bioplex	Link prediction	AUPR, Precision@k
HMNE [105]	Lazega, CKM, DBLP, C.elegans, H.genetic, PPI, and Twitter	Link prediction and node classification	F1-micro, F1-macro, and AUC
TriModel [99]	DrugBank_FDA, KEGG_MED, and Yamanishi_08	Link prediction	AUC and AUPR
FactorHNE [103]	DisGeNet, HPO and Orphanet, STRING 10	Link prediction	AUPR, AUC, Precision@K, and Recall@K
[100]	DrugBank_FDA, UNIPROT	Link prediction, node clustering	Accuracy
Hybrid model GVS [31]	GO, HPRD, CTD, HMDD and MATADOR	Link prediction	Accuracy and F1-score
DeepWalk and Node2vec [98]	DrugBank, Bio2RDF, human disease network, SIDER, KEGG, and PharmGKB	Link prediction	AUC and AUPR
Netpro2vec [92]	LFR, MREG, Kidney RNASeq, Brain fMRI COBRE, Breast RNAseq, Breast Microarray, MUTAG	Node classification	Accuracy, precision, recall, F-score, and MCC
BiLSTM [101]	Human, DUD-E, and ChEMBL	Node classification	AUC, precision, and recall
scLINE [104]	Usoskin, Li, Pollen, Patel, Darmanis, Camp, Muraro, and Petropoulos	Node clustering	DBI, NMI, ARI, Jaccard and Purity
PRD [34]	Bio2RDF, and DDI Corpus	Link prediction	AUC, AUPR
ACNE and ACNE-ST [116]	Cora, Citeseer, Wiki, and DBLP_C4	Node classification and node clustering	F1-micro and F1-macro
Pathway2vec [33]	EcoCyc, HumanCyc, AraCyc, YeastCyc, LeishCyc, and TrypanoCyc	Link prediction and node clustering	F1-micro
LPPI [28]	PPI network and GraphSAGE-PPI	Link prediction and Node Classification	Accuracy, sensitivity, precision, MCC, and AUC
MRMTI [32]	miRTarBase, miRBase, HumanNet, and biomaRt	Link prediction	AUC, AUPR, precision, recall, F1-score, and balanced accuracy
CANE [117]	Disease Encylopedia Section of XYWY.com.	Link prediction	Precision@k and recall@k

*AUPR* area under precision-recall curve, *ROC* receiver operating characteristics, *AUC* area under the curve ROC, *MCC* Matthews correlation coefficient, *DBI* Davies–Bouldin index, *NMI* normalized mutual information, *ARI* adjusted rand index, *MMR* maximum matching ratio, *MAP* mean average precision

Although Table 2 shows how graph embedding algorithms have become popular for representing biomedical data, several major limitations are apparent that limit the general applicability of graph embedding to life sciences:

- Most graph embedding algorithms have been developed to accomplish a specific task on a specific dataset, with no standards or even flexibility for incorporating other datasets. For using the same graph embedding algorithm to solve a different task, the new data set must be rewritten, thus limiting the application for other researchers.
- Shallow-embedding algorithm applications are limited in their applications, such as link prediction, node classification, and community detection tasks. More complex problems such as graph matching, subgraph matching, and calculating the maximum common subgraphs require more complex models requiring combinatorial optimization (graph theory). Furthermore, these problems are solved through representation learning (deep learning). However, most deep-learning graph embedding techniques are not deterministic because they use probabilities to perform their tasks, yielding similar, but not identical results for different runs.
- Loss of structural information: graph embedding methods typically aim to preserve the proximity of nodes based on their graph structure. However, they may lose certain structural information during the embedding process. For instance, (i) higher-order relationships within the graph may not be accurately captured. Furthermore, (ii) graph embeddings may not effectively leverage node attributes or features. Node attributes (metadata) can provide valuable information in life sciences, such as measurement conditions. It may be computationally expensive to maintain graph embeddings for (iii) dynamic data sets where nodes and edges are frequently added, removed, or modified (due to experimental conditions).
- Interpretability: The interpretability of graph embeddings can be more challenging compared to other clustering techniques, as it is often difficult to interpret the specific features or relationships each dimension captures.

Addressing these limitations is an active area of research, and researchers continue to develop new techniques and algorithms to enhance the performance and versatility of graph embedding methods to make them more applicable to life-science research questions.

## Conclusion

As can be easily appreciated from the by far not exhaustive list of discussed algorithms for graph embedding in this review, there is currently not yet a gold standard for graph embedding for biological data emerging that can provide reliable data for biologists and serve as a reference point for future developments of in the field. So far, the presented applications for graph embedding on biological data have all been developed for the specific data sets at hand. All these studies have thus mainly remained theoretical, focusing on the development of computational techniques rather than taking the interpretation of the data to the identification of novel biology or drug developments. Yet, with the ever-growing datasets available to life science researchers, the community needs novel tools to understand better the underlying biological processes. Given their nature of reducing the dimensionality of complex data, graph embedding algorithms are an exciting and novel tool for extracting novel insight from large biological datasets (Table 2). We envision that graph embedding will become an essential tool aiding hypothesis generation leading to novel biological discoveries.

Specifically, graph embedding techniques hold significant potential in various biological and biomedical research fields. In the context of the drug–disease association (DDA), disease-gene association (DGA), drug–target interaction (DTI), protein–protein interaction (PPI), and drug–drug interaction (DDI) (Table 2), graph embedding methods can provide valuable insights and aid in understanding complex relationships. By representing drugs, diseases, genes, targets, and proteins as nodes in a graph and capturing their interactions as edges, graph embedding algorithms can (i) infer novel insight into a biological system based on information about its elements (i.e., link prediction), (ii) classify the relevance of biological elements (e.g., proteins, metabolites, etc.) and their interactions within a system (i.e., node classification), and (iii) identify a phenotype or physiology of interest based on the networks formed by their elements (i.e., node clustering).

Furthermore, with the help of low-dimensional representations obtained using graph-neural networks (GNN) algorithms, it is possible to encode the underlying relationships and functional associations to find similarities between individuals sharing the same condition (e.g., graph matching or subgraph matching). These low-dimensional embeddings can then be leveraged to gain an understanding of the underlying molecular events occurring within the biological system (i.e., molecular phenotype characterization).

Hence, the ability to integrate multiple data sources, such as genomic, transcriptomic, proteomic, metabolomic, and clinical data, further enhances the predictive power and potential impact of graph embedding techniques, mainly in the field of personalized medicine, paving the way for improved disease management, identifying potential therapeutic targets, elucidating underlying molecular mechanisms, and exploring drug synergy or adverse interactions.

In conclusion, this increased predictive power gained by using graph embedding techniques on biological data will allow life-science researchers to conduct more targeted experiments by extracting novel unseen links. Developing applications will require substantial further research on the bioinformatic side to identify the most promising approaches to be applied to specific types of datasets, as well as thorough experimental validation of the generated outputs. Despite posing a challenging problem to either field, the rapid rise of AI tools in our everyday life as a researcher will certainly fuel interest in incorporating novel AI-based analysis methods on high dimensional biological data. Therefore, we anticipate that graph embedding applications will soon be invaluable in the broader life science community.

## Data Availability

Not Applicable.
